# The virtual Morris water maze for cognitive function assessment in adolescents with type 1 diabetes

**DOI:** 10.1007/s00125-025-06598-x

**Published:** 2025-11-17

**Authors:** Hussein Zaitoon, Liat Perl, Eyal Cohen-Sela, Asaf Oren, Yael Lebenthal, Avivit Brener

**Affiliations:** 1 Gray Faculty of Medical and Health Sciences, Tel Aviv University, Tel Aviv, Israel; 2 The Institute of Pediatric Endocrinology, Diabetes and Metabolism, ‘Dana-Dwek’ Children’s Hospital, Tel Aviv Sourasky Medical Center, Tel Aviv, Israel

**Keywords:** Cognitive function, Glycaemic management, Spatial navigation, Type 1 diabetes, Virtual Morris water maze

## Abstract

**Aims/hypothesis:**

Neuropsychological assessments and neuroimaging techniques have indicated impaired spatial working memory in adolescents with type 1 diabetes. We investigated the influence of diabetes-related factors on their spatial navigation performance.

**Methods:**

Spatial navigation performance on the virtual Morris water maze task (vMWMT) was evaluated in adolescents with type 1 diabetes and compared with that of healthy control adolescents of similar age and sex. Collected data on diabetes-related variables included disease duration, diabetic ketoacidosis (DKA) at diagnosis and continuous glucose monitoring (CGM) metrics during the 2 weeks preceding the assessment, with focus upon nocturnal values measured during the night before testing. Time to first move, time to platform and path length were measured in visible and hidden platform vMWMT stages.

**Results:**

The 74 study participants (age 15.6 ± 3.1 years, 45 boys) with type 1 diabetes demonstrated sex-specific patterns of spatial navigation, comparable with those observed in the healthy control group. Both the boys and girls had longer time to first move than the control groups in the visible platform stage, which assessed motor control (*p*=0.036 and *p*=0.002, respectively). Test outcomes did not differ between the participants with and without type 1 diabetes in the hidden platform stages, which assessed spatial learning and memory. However, linear regression models adjusted for sex, age and DKA at diagnosis found that diabetes duration (β=0.464, *p*<0.001) independently predicted longer time to platform (*R*^2^=0.396, *p*=0.003), while nocturnal time spent in marked hypoglycaemia (β=0.397, *p*=0.002) predicted longer path length (*R*^2^=0.206, *p*=0.017).

**Conclusions/interpretation:**

Spatial navigation performance in adolescents with type 1 diabetes is influenced by both disease duration and recent glycaemic control. Glycaemic excursions, especially during the night, were shown to impair performance.

**Graphical Abstract:**

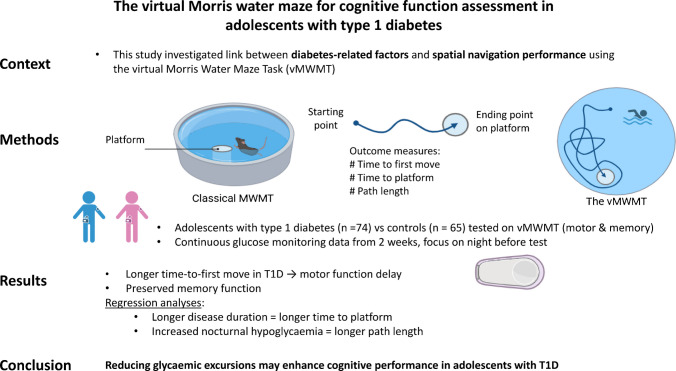

**Supplementary Information:**

The online version of this article (10.1007/s00125-025-06598-x) contains peer-reviewed but unedited supplementary material.


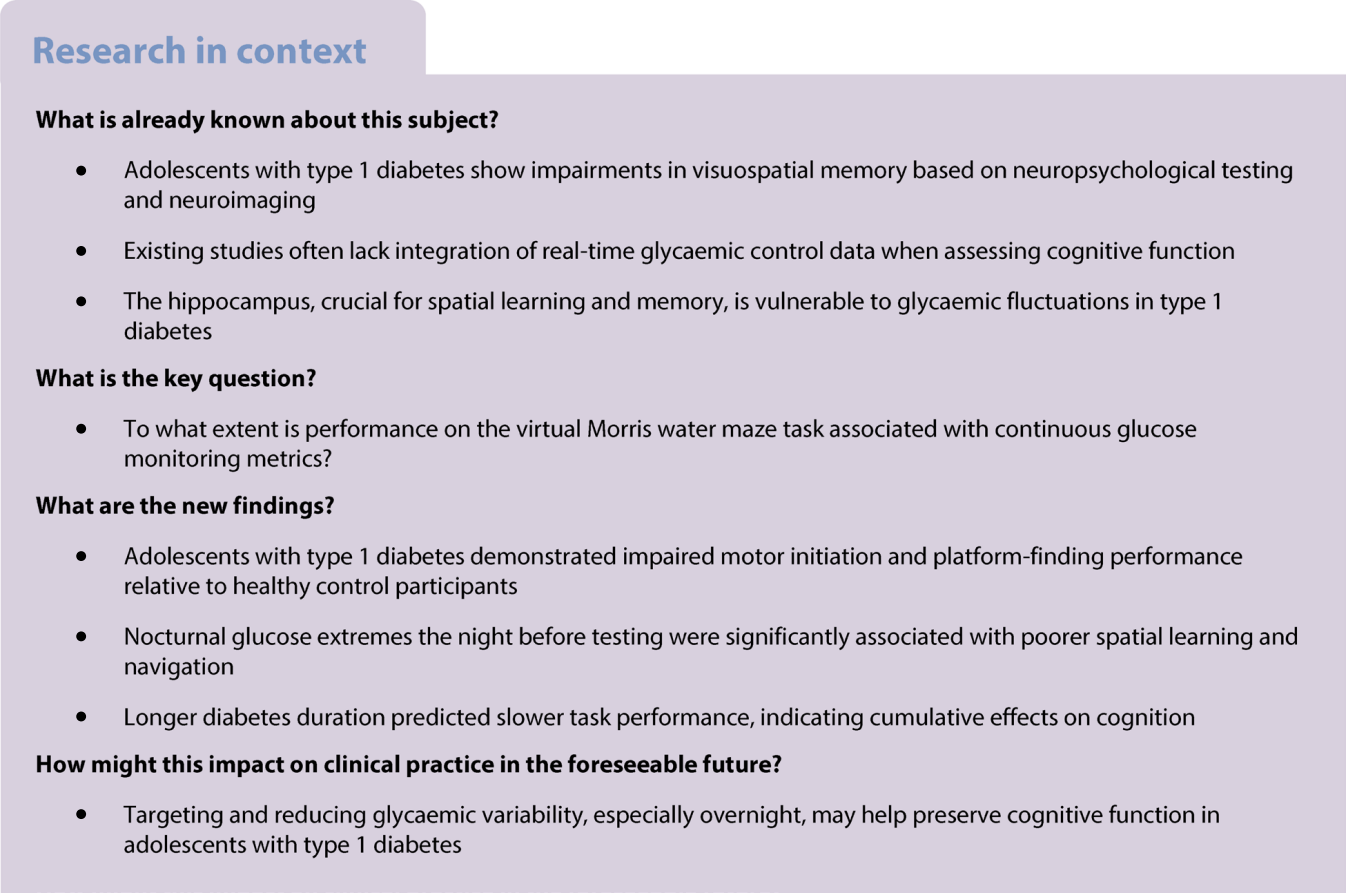


## Introduction

Accumulating evidence points to mild-to-moderate cognitive deficits and compromised academic achievement in adolescents with type 1 diabetes [1, 2]. In individuals with type 1 diabetes, extreme glycaemic fluctuations, particularly hypoglycaemia and, to a lesser extent, hyperglycaemia, may be associated with declines in cognitive functions [3, 4]. Distinct central neuropathic alterations in the hippocampus, a region critical for memory formation, attention and spatial navigation, were reported in individuals with type 1 diabetes [5]. Rodent models of diabetes revealed hippocampal electrophysiological abnormalities and related deficits in learning, memory and cognition [6]. These findings include impaired synaptic plasticity, deficits in long-term potentiation and long-term depression [7, 8], leading to impairments in spatial memory tasks [9].

Few studies have examined cognitive functioning in adolescents with type 1 diabetes, and those that have, often present important limitations. Notably, most have not considered real-time glycaemic control [10, 11]; a critical factor given the dynamic impact of glucose fluctuations on brain function. Technology-driven therapies for diabetes, including continuous glucose monitoring (CGM) and open-source automated insulin delivery systems (OS-AIDs), have revolutionised both diabetes care and research [12, 13]. In addition, previous studies have primarily relied on neuropsychological testing and neuroimaging, which, while informative, may not adequately capture how cognitive skills are applied in real-life contexts. Spatial navigation tasks offer a more ecologically valid alternative, as they require the integration of multiple cognitive domains, including learning, memory, attention and executive function. As such, they provide an ideal model for assessing subtle cognitive effects of glycaemic variability in everyday conditions.

The virtual Morris water maze task (vMWMT) provides a validated tool for assessing spatial navigation as an indicator of multi-dimensional cognitive performance [14]. In this study, we examined the contribution of diabetes-related variables towards performance in the vMWMT among adolescents with type 1 diabetes.

## Methods

### Study design and participants

This study included adolescents aged 10–20 years who had been diagnosed with type 1 diabetes for longer than 1 year. Participants were recruited during their routine diabetes clinic visits at a tertiary medical centre. Those with a history of neurological and/or psychiatric disorders and those diagnosed with developmental delays, as well as those unable to understand or complete the water maze task, were excluded.

At our centre, the standard care practice for monitoring and follow-up of type 1 diabetes patients involves quarterly clinic visits, with input from a multidisciplinary medical team that includes diabetes-specialised nurses, dietitians, psychosocial professionals and paediatric endocrinologists. During these visits, vital signs and anthropometric measurements (height, weight and BMI) are recorded. Capillary HbA_1c_ levels are determined using the DCA Vantage Analyzer range 4 mmol/mol [2.5%] to 129 mmol/mol [14%]). Data from glucometers, CGM devices and insulin pumps are downloaded, providing the basis for management decisions.

Eligible participants and their parents received a comprehensive explanation of the study’s objectives, task requirements and estimated duration. Written informed assent was obtained from participants and consent from their guardians. The study was approved by the institutional review board (TLV-0436-23). The control group comprised healthy volunteers in similar age recruited by the research team at the University of Haifa, with approval from the Department of Psychology Ethics Committee (no. 064/19).

### Water maze task

The vMWMT is a computerised, human-adapted version of the classic Morris water maze, validated as a reliable tool for assessing spatial cognition in children and adolescents [14]. Participants navigate in a virtual pool or arena, aiming to locate a hidden platform using distal visual cues. This task specifically engages hippocampal-dependent visuospatial memory, requiring individuals to encode, retain and retrieve spatial information about the environment and the platform’s location.

**Fig. 1:**
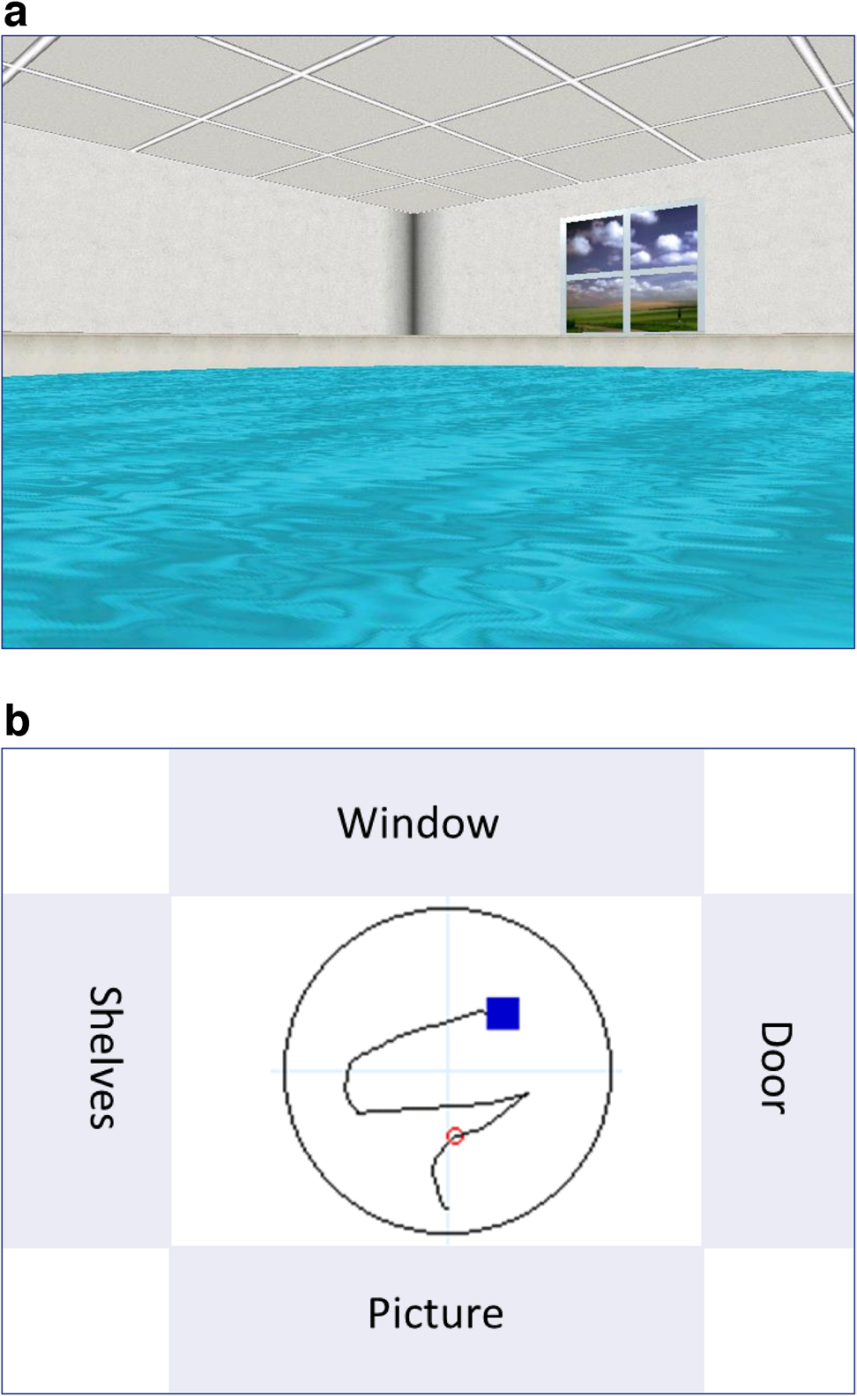
(**a**) First-person view of the virtual environment as seen by participants on a computer screen during the vMWMT. (**b**) Top-down overview of the virtual arena. Each wall displays a distinct visual cue to assist spatial orientation and target localisation. The black circle outlines the pool boundary, and the blue square marks the hidden platform. The black trace-line depicts the participant’s actual navigation route in the trial shown, while the red circle highlights the shortest trajectory achieved by the same participant across multiple attempts

A 15.6 inch laptop computer monitor was used to display the virtual environment generated by the vMWMT software version 1.10 (Neuro Investigations, Lethbridge, AB, Canada). The task presents a square room with a round pool filled with water (Fig. 1a). Four rectangular abstract paintings were placed on the room’s walls as location cues, differing in shape, colour and position (Fig. 1b). Participants viewed the room from a point above the waterline and navigated by means of keyboard arrow keys. Movement in the backward or vertical direction was not possible. The four stages of the vMWMT, described below, were as follows: (1) the exploration stage; (2) the visible platform stage; the hidden platform stage; and (4) the probe stage. The following primary measures were collected during both visible and hidden phases: time to first move, defined as the duration between trial beginning and the initiation of movement; time to platform, defined as the time taken to reach the platform; path length, representing the total distance covered relative to the pool diameter; and the total time spent in the correct quadrant of the pool. The entire task duration was 10–15 min. All stages were completed in a single session without any rest intervals. The probe trial (memory retrieval) was conducted immediately following the hidden platform trials, allowing for assessment of short-term spatial memory.

#### Exploration stage

The exploration stage was a 30 s training phase to allow all participants to become familiar with the virtual environment and to practice swimming, without measurements. No platform was present during this phase. Participants could ask the examiner questions before the main task began.

#### Visible platform stage

For motor control condition, initially, the participants completed four trials with a visible platform in the pool to become familiar with the mechanics of the task. They were instructed to swim toward the platform as quickly as possible.

#### Hidden platform stage

To assess spatial learning, the test continued with four blocks, each consisting of four trials with a hidden platform, for a total of 16 trials. The virtual environment featured visual cues that were different from those in the training phase. The hidden platform was located at the same spot in the northeastern quadrant of the pool in all trials. It was submerged beneath the surface and thus invisible to participants. Starting locations for each trial were randomly assigned from the four corners of the pool. If the participants could not find the platform within 60 s, the platform was made visible and they were instructed to swim towards it.

#### Probe stage

In this last trial, the platform was removed from the pool. During this session, spatial memory retrieval was assessed by measuring the percentage of time each participant spent in the quadrant where the platform had previously been located. A higher percentage indicated a more accurate recall of the platform’s location.

### Data collection and variables

Data extracted from medical records included sociodemographic characteristics (sex, age and socioeconomic position [SEP]), determined by using residential address data and referencing the 2015 classification system from the Israel Central Bureau of Statistics [15], which ranks neighbourhoods into deciles (1–10). A SEP score ranging from –2.797 to 2.590 reflects a composite of 14 demographic, educational, income and employment indicators, with higher scores indicating more favourable conditions, medical history (thyroid disease, coeliac disease, attention deficit hyperactivity disorder), diabetes-related characteristics and clinical variables (anthropometric measurements, BP measurements and Tanner pubertal stage) recorded during the clinic visit during which the task was performed. Anthropometric measurements of height and BMI were converted to sex- and age-appropriate *z* scores based upon Center of Disease Control and Prevention growth charts.

### Diabetes-related characteristics and glycaemic control

The retrieved data also included age at diagnosis, which was used to calculate disease duration, and diabetes-related characteristics at onset (HbA_1c_ levels and presence and severity of diabetic ketoacidosis [DKA]). Current diabetes-related characteristics were recorded, including CGM usage and insulin therapy modality (multiple daily injections [MDI], continuous subcutaneous insulin infusion via insulin pump alone or as part of automated insulin delivery [AID] systems). AID systems included either commercial hybrid closed-loop systems integrating CGM and insulin pumps with proprietary algorithms for automated delivery, or configurations using CGM and pumps in conjunction with open-source, user-developed algorithms for AID systems designed to achieve similar outcomes [16].

Glycaemic control parameters included HbA_1c_ measurements, continuous CGM metrics and blood glucometer data for the 2 weeks preceding the vMWMT, and the current insulin dose (U/kg per day). HbA_1c_ values obtained between 1 year after diagnosis and the date of task performance were analysed. CGM data were extracted from the ambulatory glucose profile report, which included CGM active time, mean glucose levels, glucose SD, glucose management indicator (GMI), CV and per cent of time spent in various glucose ranges. Glucose ranges were recorded according to international CGM interpretation guidelines [17], including time in range (3.9–10.0 mmol/l [70–180 mg/dl]), time in hypoglycaemia (<3.9 mmol/l [<70 mg/dl]), time in marked hypoglycaemia (<3.0 mmol/l [<54 mg/dl]), time in hyperglycaemia (>10.0 mmol/l [>180 mg/dl) and time in severe hyperglycaemia (>13.9 mmol/l [>250 mg/dl]).

CGM data from the 8 h period between 00:00 hours and 08:00 hours were analysed in order to evaluate nocturnal glycaemic patterns on the night prior to the vMWMT. Those data included the minimum and maximum glucose levels, as well as the percentage of time spent in marked hypoglycaemia (<3.0 mmol/l [<54 mg/dl]) and in severe hyperglycaemia (>13.9 mmol/l [>250 mg/dl]). All CGM traces were visually reviewed to identify and exclude compression artefacts. Events were considered valid if they lasted ≥15 min and followed a physiological pattern (gradual decline and recovery) consistent with true hypoglycaemia.

### Statistical analysis

All statistical analyses were performed using IBM SPSS Statistics for macOs, Version 29.0 (IBM Corp., Armonk, NY, USA, 2023). The Kolmogorov–Smirnov or Shapiro–Wilk tests were used to assess the normality of continuous data. Data are expressed as mean ± SD for normally distributed variables and as median with IQR for non-normally distributed variables. Repeated-measures ANOVA analyses, stratified by sex, were conducted to evaluate group (type 1 diabetes vs control group) and time (change between four test blocks) effects and the interaction between group and time for three task performance measures (time to first move, time to platform and path length). In addition, comparisons between task performance measures (average of four blocks) were then conducted between the boys and girls with type 1 diabetes and their healthy control counterparts by means of independent sample *t* tests. Categorical variables were compared by means of χ^2^ tests or Fisher’s exact tests, as appropriate. Correlations between participants’ clinical and glycaemic characteristics and performance on the vMWMT, including both the hidden platform phase (time to first move, time to platform and path length) and the probe phase (time spent in the correct quadrant), were evaluated with Pearson or Spearman correlation coefficients, depending upon the normality of variable distributions. To account for multiple comparisons, we applied a Bonferroni correction.

The following variables were included in the correlation analysis: BMI *z* score; age at type 1 diabetes diagnosis; diabetes duration; HbA_1c_ level at type 1 diabetes diagnosis; HbA_1c_ level at the current visit; and CGM metrics during the 2 weeks preceding the task and during the night before testing.

Multiple linear regression analyses were conducted to identify independent predictors of vMWMT performance during the hidden platform phase. Separate models were constructed for each outcome variable. All models were adjusted for sex, age and the presence of DKA at diagnosis, and included the following variables: sex; age; duration of type 1 diabetes; DKA at diagnosis; HbA_1c_ at diagnosis and at the current visit; and CGM metrics during the 2 weeks prior to and the night before task performance. A *p* value of ≤0.05 was considered statistically significant.

## Results

### Participant characteristics

A total of 74 adolescents (45 boys, 60.8%) aged 15.6 ± 3.1 years with type 1 diabetes participated in the study (Table 1). They had been diagnosed with diabetes at a mean age of 8.9 ± 3.8 years, 29 (39.2%) presented with DKA, and the group’s median diabetes duration was 5.9 (IQR 3.8–9.0) years. The median HbA_1c_ at the time of task performance was 53 (IQR 46–60) mmol/mol (7.0 [IQR 6.4–7.6]%). The mode of insulin therapy included MDI in 19 (25.7%) adolescents, insulin pump therapy alone in 33 (44.6%), and as part of an AID system in 22 (29.7%). The pump types were Omnipod (*n*=37, 50%), Medtronic G640 (*n*=2, 2.7%), Medtronic G780 (*n*=15, 20.3%), Tandem t:slim X2 (*n*=1, 1.35%) and Accu-Chek (*n*=1, 1.35%). Almost all the participants (*n*=71, 95.9%) used CGM, the most common of which was Dexcom G6/7 (*n*=53, 71.6%), followed by Guardian (*n*=17, 23%) and Libre (*n*=1, 1.35%). Insulin therapy and pump types had been selected by participants and their parents based upon individual preferences and the reimbursement coverage provided by their health maintenance organisation within the framework of the national health basket.

**Table 1:** Sociodemographic and diabetes-related characteristics of participants

Variable	Values
No. of participants	74
Male sex, *n* (%)	45 (60.8)
Age, years	15.6 ± 3.1
SEP cluster^a^	8 (5.0–9.0)
SEP index	1.266 (0.043–1.833)
Comorbid conditions, *n* (%)	
Coeliac disease	6 (8.1)
Autoimmune thyroid disease	4 (5.4)
Attention deficit hyperactivity disorder	7 (9.5)
BP, mmHg	
Systolic BP	111.7 ± 8.5
Diastolic BP	68.9 ± 7.5
BMI *z* score	0.3 ± 1.1
Pubertal status, *n* (%)^b^	
Prepubertal	4 (5.4)
In puberty	21 (28.3)
Fully pubertal	49 (66.2)
Diabetes-related characteristics	
Age at diagnosis, years	8.9 ± 3.8
DKA at diagnosis, *n* (%)	29 (39.2)
HbA_1c_ at onset, mmol/mol	102 ± 25
HbA_1c_ range at onset, mmol/mol	53–165
HbA_1c_ at onset, %	11.5 ± 2.3
HbA_1c_ range at onset, %	7–17.2
Diabetes duration, years	5.9 (3.8–9.0)
HbA_1c_ at task performance, mmol/mol	53 (46–60)
HbA_1c_ range at task performance, mmol/mol	36–85
HbA_1c_ at task performance, %	7.0 (6.4–7.6)
HbA_1c_ range at task performance, %	5.4–10.0
Mean HbA_1c_ (from 1 year after diagnosis until task performance day), mmol/mol	56 (51–61)
Mean HbA_1c_ (from 1 year after diagnosis until task performance day), %	7.2 (6.8–7.7)
HbA_1c_ mean SD, mmol/mol %	5 (4–7)
HbA_1c_ mean SD, %	0.5 (0.4–0.7)
No. of HbA_1c_ measurements (from 1 year after diagnosis until task performance day)	18.1 ± 9.0

Data are presented as *n* (%), mean ± SD or median (IQR)

^a^Sociodemographic position was determined by cluster of localities of residence, ranging from 1 to 10, with 1 being the lowest rating and 10 the highest

^b^Pubertal stage was graded according to Marshall and Tanner: ‘prepubertal’ is defined as Tanner stage 1; ‘in puberty’ as Tanner stages 2–4; and ‘fully pubertal’ as Tanner stage 5

The control group included 65 healthy adolescents (37 girls [57%]), with no history of neurological, psychiatric or other chronic medical conditions. The boys’ mean age was 16.3 ± 1.1 years and the girls’ mean age was 15.9 ± 1.3 years (*p*=0.195).

### CGM metrics during the 2 weeks prior to competing the vMWMT

The average mean glucose level for the entire type 1 diabetes cohort was 9.08 ± 1.7 mmol/l (163.5 ± 30.2 mg/dl), with a mean SD of 62.7 ± 18.1 and a mean GMI of 7.2 ± 0.7. The median time in range was 64.5 (IQR 49.5–75.2)%. Comparisons between CGM metrics of boys and girls revealed no significant sex differences in mean glucose levels or time spent in glycaemic ranges (Table 2). However, girls exhibited significantly higher glycaemic variability, as indicated by a greater CV (*p*=0.036). This difference did not remain significant after Bonferroni correction.

**Table 2:** Comparison of diabetes-related characteristics and CGM metrics between boys and girls with type 1 diabetes in the 2 week period prior to the vMWMT task performance

Variable	Boys	Girls	*p* value
*n* (%)	45 (60.8)	29 (39.2)	
Age, years	15.8 ± 2.9	15.4 ± 3.5	0.644
Diabetes-related characteristics			
DKA at onset, *n* (%)			0.835
No	26 (57.8)	19 (65.5)	
Mild	8 (17.8)	5 (17.2)	
Moderate	6 (13.3)	2 (6.9)	
Severe	5 (11.1)	3 (10.3)	
HbA_1c_ at onset, mmol/mol	103 ± 23	98 ± 29	0.451
HbA_1c_ at onset, %	11.6 ± 2.1	11.2 ± 2.6	
Diabetes duration, years	6.4 (3.5–9.0)	5.5 (4.4–12.3)	0.833
HbA_1c_ at task performance, mmol/mol	52 (46–57)	54 (52–62)	0.118
HbA_1c_ at task performance, %	6.85 (6.4–7.3)	7.10 (6.9–7.8)	
Mean HbA_1c_ (from 1 year after diagnosis until task performance day), mmol/mol	55 (51–60)	56 (52–62)	0.223
Mean HbA_1c_ (from 1 year after diagnosis until task performance day), %	7.17 (6.7–7.6)	7.28 (6.9–7.9)	
HbA_1c_ SD (from 1 year after diagnosis until task performance day), mmol/mol	6 (4–8)	6 (4–9)	0.864
HbA_1c_ SD (from 1 year after diagnosis until task performance day), %	0.56 (0.4–0.7)	0.52 (0.4–0.8)	
Insulin delivery mode, *n* (%)			
Multiple daily insulin injections	12 (26.7)	7 (24.1)	0.965
Sensor-augmented pump	20 (44.4)	13 (44.8)	
Sensor-integrated pump	13 (28.9)	9 (31.0)	
CGM metrics			
Time CGM active, %	94.2 ± 6.5	91.6 ± 12.1	0.250
Mean glucose, mmol/l [mg/dl]	8.99 ± 1.58 [162.0 ± 28.5]	9.20 ± 1.87 [165.7 ± 33.7]	0.623
Glucose SD, mmol/l [mg/dl]	3.31 ± 1.05 [59.7 ± 18.9]	3.74 ± 0.91 [67.4 ± 16.3]	0.085
CV, %	37.5 ± 6.5	40.9 ± 6.4	0.036^a^
GMI, %	7.2 ± 0.7	7.3 ± 0.8	0.509
Time in glucose ranges, %			
Time in range, 3.9–10.0 mmol/l (70–180 mg/dl)	65 (55.0–80.0)	64 (47.8–74.0)	0.399
Time at <3.0 mmol/l (<54 mg/dl)	0 (0.0–1.0)	0 (0.0–2.0)	0.460
Time at <3.9 mmol/l (<70 mg/dl)	1 (1.0–4.0)	3 (1.5–5.0)	0.079
Time at >10.0 mmol/l (>180 mg/dl)	21 (15.0–26.0)	20 (15.8–25.5)	0.796
Time at >13.9 mmol/l (>250 mg/dl)	8 (2.4–16.0)	10 (4.0–22.5)	0.386
Nocturnal glucose metrics in the previous night			
Minimum glucose value, mmol/l [mg/dl]	4.1 (3.3–5.7) [74 (59.0–102.0)]	4.7 (3.3–5.6) [85 (60.2–100.5)]	0.796
Maximum glucose value, mmol/l [mg/dl]	11.3 (9.4–15.0) [204 (169.0–269.5)]	11.2 (8.5–16.5) [202 (152.2–296.2)]	0.972
Nocturnal glucose preceding the task performance, mmol/l [mg/dl]	6.8 (4.3–8.3) [122 (77.0–148.5)]	7.2 (3.6–10.5) [130 (64.5–188.2)]	0.917
Time at <3.9 mmol/l (<70 mg/dl), %	0 (0.0–3.5)	0 (0.0–7.7)	0.957
Time at <3.0 mmol/l (<54 mg/dl), %	0 (0.0–0.0)	0 (0.0–0.0)	0.393
Time at >10.0 mmol/l (>180 mg/dl), %	10.5 (0.0–33.5)	10.9 (0.0–39.9)	0.949

Data are expressed as *n* (%), median (IQR) or mean ± SD

^a^Not statistically significant after Bonferroni correction

*p*≤0.05 indicates statistical significance; χ^2^ tests were performed to compare categorical variables between groups, and the Mann–Whitney test was performed to compare linear variables with skewed distribution

### Results of the vMWMT

#### Visible platform stages: motor control condition

On average, boys with type 1 diabetes showed a significantly longer time to first move compared with healthy controls (5.03 ± 2.83 vs 3.96 ± 1.45 s, *p*=0.036) and a significantly longer time to platform (12.91 ± 8.31 vs 8.74 ± 3.32 s, *p*=0.004). Path length was also significantly longer among boys with type 1 diabetes (0.50 ± 0.17 vs 0.44 ± 0.07 m, *p*=0.025). Girls with type 1 diabetes also demonstrated a significantly longer time to first move compared with healthy controls (7.96 ± 6.86 vs 4.77 ± 1.69 s, *p*=0.02) and had a significantly longer time to platform (22.26 ± 23.90 vs 9.81 ± 2.48 s, *p*=0.009). No differences in path length emerged when comparing girls with type 1 diabetes and their control counterparts (0.85 ± 1.82 vs 0.43 ± 0.06 m, *p*=0.222).

Boys with type 1 diabetes exhibited both significantly shorter time to first move compared with girls with type 1 diabetes (5.03 ± 2.83 vs 7.96 ± 6.86 s, *p*=0.036) and shorter time to platform (12.91 ± 8.32 vs 22.26 ± 23.90 s, *p*=0.05). No sex-based differences were found in path length (0.50 ± 0.17 vs 0.85 ± 1.82 m, *p*=0.309). Similar sex-based differences were observed in healthy control adolescents, with boys demonstrating shorter time to first move and time to platform than girls.

#### Hidden platform stages: spatial learning

**Fig. 2:**
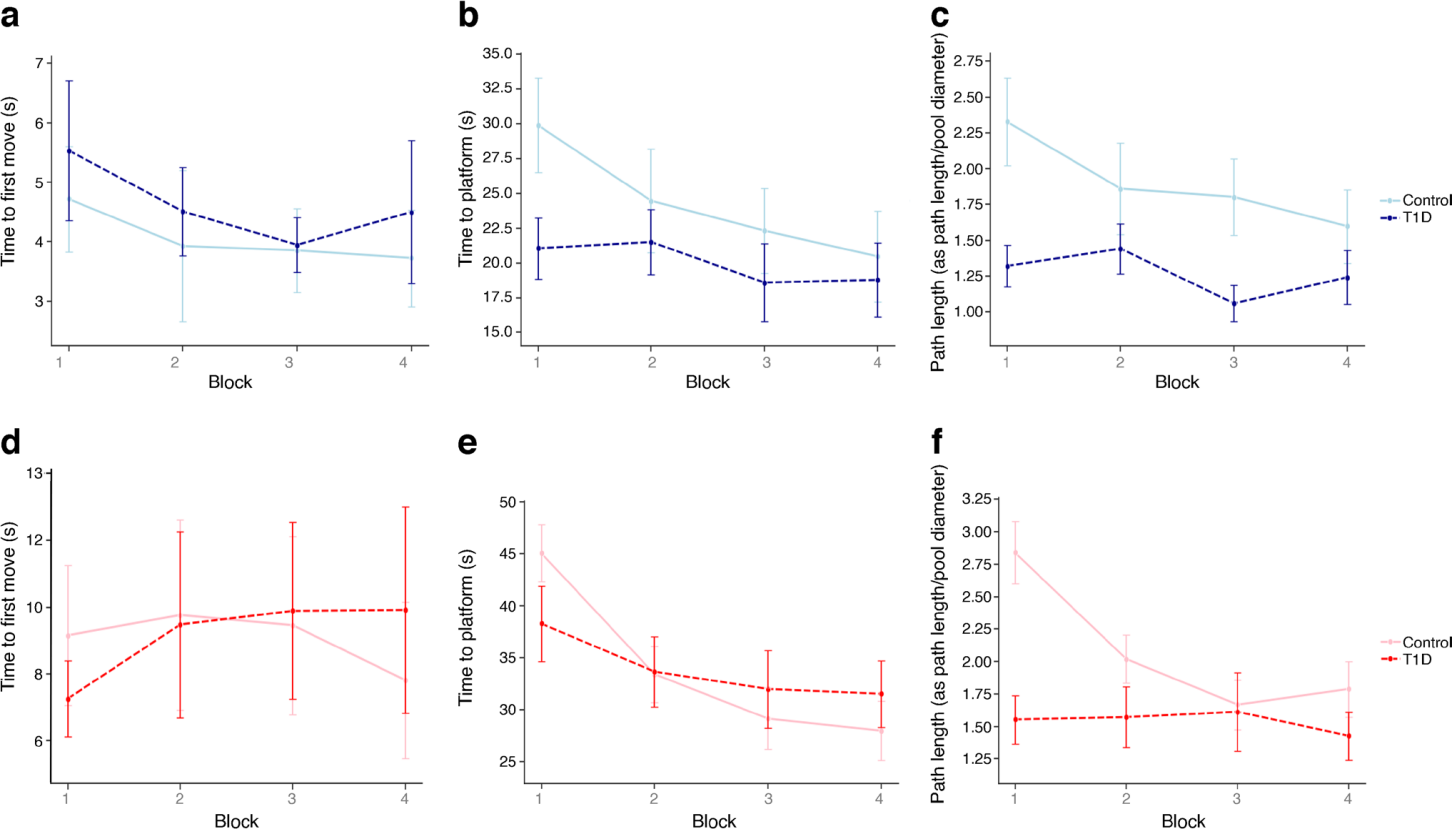
Average task performance variables (in 4 blocks) in adolescents with type 1 diabetes and healthy control adolescents, stratified by sex. (**a**–**c**) Time to first move (**a**), time to reach the hidden platform (time to platform) (**b**) and path length to reach the hidden platform (**c**) in adolescent boys with type 1 diabetes compared with the non-diabetic control group. (**d**–**f**) Time to first move (**d**), time to platform (**e**) and path length (**f**) in adolescent girls with type 1 diabetes compared with the non-diabetic control group. Data are shown as mean ± SD. Group sizes were: boys with type 1 diabetes (*n*=45), girls with type 1 diabetes (*n*=29), boys in the control group (*n*=37), and girls in the control group (*n*=28)

The results of repeated-measures ANOVA analyses, stratified by sex, conducted to evaluate the effects of group (type 1 diabetes vs healthy control) and time (change between four test blocks), and the interaction between group and time, for three task performance variables (time to first move, time to platform and path length) are presented in Table 3 and Fig. 2. Average time to first move and time to platform among the boys with type 1 diabetes were comparable with those of their healthy control counterparts (time to first move 4.62 ± 4.29 vs 4.06 ± 3.23 s, *p*=0.525; time to platform 20.40 ± 14.33 vs 24.28 ± 16.15 s, *p*=0.303). They did, however, have a shorter path length compared with their control group (1.30 ± 0.89 vs 1.90 ± 1.31 m, *p*=0.04). No significant differences were observed between the girls with type 1 diabetes and the healthy control girls in time to first move (9.08 ± 12.20 vs 9.05 ± 14.62 s, *p*=0.992); time to platform (34.10 ± 16.65 vs 33.92 ± 15.32 s, *p*=0.964); or path length (1.57 ± 1.07 vs 2.08 ± 1.08 m, *p*=0.061).

**Table 3:** Repeated-measures ANOVA of task performance variables stratified by sex, evaluated group (type 1 diabetes vs control) and time effects, and the interaction between group and time

Task performance variable	Block 1	Block 2	Block 3	Block 4	*p* value for time	*p* value for group	*p* value for interaction
Boys							
Time to first move, s							
Type 1 diabetes	5.6 ± 1.0	4.5 ± 0.9	3.9 ± 0.5	4.5 ± 1.0	0.441	0.547	0.928
Control	4.7 ± 1.3	3.9 ± 1.1	3.9 ± 0.6	3.7 ± 1.3			
Time to platform, s							
Type 1 diabetes	20.4 ± 2.4	20.5 ± 2.5	18.6 ± 2.7	18.8 ± 2.6	0.008^a^	0.183	0.129
Control	29.9 ± 3.0	24.5 ± 3.2	22.3 ± 3.3	20.5 ± 3.3			
Path length, m							
Type 1 diabetes	1.3 ± 0.2	1.4 ± 0.2	1.1 ± 0.2	1.2 ± 0.2	0.032^b^	0.009	0.071
Control	2.3 ± 0.2	1.9 ± 0.3	1.8 ± 0.2	1.6 ± 0.2			
Girls							
Time to first move, s							
Type 1 diabetes	7.4 ± 2.0	9.7 ± 3.1	10.1 ± 2.9	9.9 ± 2.9	0.371	0.289	0.947
Control	9.2 ± 1.7	9.8 ± 2.7	9.5 ± 2.5	7.8 ± 2.5			
Time to platform, s							
Type 1 diabetes	38.1 ± 3.4	32.5 ± 3.2	31.1 ± 3.6	31.5 ± 3.2	<0.001^a^	0.878	0.015
Control	45.1 ± 3.0	33.4 ± 2.8	29.2 ± 3.1	28.0 ± 2.8			
Path length, m							
Type 1 diabetes	1.5 ± 0.2	1.4 ± 0.2	1.5 ± 0.3	1.4 ± 0.2	<0.001^a^	0.017	<0.001
Control	2.8 ± 0.2	2.0 ± 0.2	1.7 ± 0.2	1.8 ± 0.2			

Data are expressed as estimated mean ± SE

^a^Significant difference was found between block 1, and blocks 2, 3, 4

^b^Significant difference was found between block 1, and blocks 3, 4

*p*≤0.05 was considered significant

A sex-based comparison revealed that boys with type 1 diabetes had significantly shorter time to platform compared with girls with type 1 diabetes (20.40 ± 14.33 vs 34.10 ± 16.65 s, *p*<0.001) while no significant sex-based differences were found in time to first move (4.62 ± 4.29 vs 9.08 ± 12.20 s, *p*=0.067) or path length (1.30 ± 0.89 vs 1.57 ± 1.07 m, *p*=0.249).

Analysis of movement initiation in the hidden stages revealed that lower minimal nocturnal glucose values were associated with longer time to first move (*r*=0.267, *p*=0.035, not significant after Bonferroni correction) (ESM Table 1). The mean time to platform was positively correlated with BMI *z* score (*r*=0.281, *p*=0.015) and with type 1 diabetes duration (*r*=0.395, *p*=0.002), and inversely correlated with CGM active time (*r*=−0.255, *p*=0.034 not significant after Bonferroni correction). None of the remaining correlations with test time measurements reached a level of significance. Increased HbA_1c_ SD (*r*=–0.295, *p*=0.011), higher CV (*r*=0.253, *p*=0.037, not significant after Bonferroni correction) and the proportion of nocturnal time spent in marked hypoglycaemia (*r*=0.394, *p*<0.001) were correlated with longer path length in the virtual distance measurements in the hidden stages.

Multiple regression analyses are presented in Table 4. The finding for the model for time to platform was significant (*R*^2^=0.396, *p*=0.003), with female sex (β=0.475, *p*<0.001) and longer diabetes duration (β=0.464, *p*<0.001) emerging as significant predictors of increased time to platform. The findings for the model predicting path length were significant (*R*^2^=0.206, *p*=0.017), with the time spent in marked hypoglycaemia during the 2 weeks preceding task performance emerging as the only significant predictor of longer path length (β=0.397, *p*=0.002). No other variables reached a level of significance in this model.

**Table 4:** Linear regression models evaluating the association between task performance and other variables

Variable	SE	β	*p* value
Time to first move			0.256^a^
Sex	2.108	0.306	0.040
Age	0.364	−0.068	0.667
Type 1 diabetes duration	0.062	0.155	0.305
DKA at presentation	1.959	−0.045	0.753
Time at <3.0 mmol/l (<54 mg/dl)	1.105	−0.123	0.470
Time in range, 3.9–10.0 mmol/l (70–180 mg/dl)	0.156	0.019	0.959
Time at >10.0 mmol/l (>180 mg/dl)	0.198	0.121	0.543
CV	0.204	−0.290	0.111
Time to platform			0.003^a^
Sex	4.295	0.475	<0.001
Age	0.741	−0.069	0.615
Type 1 diabetes duration	0.126	0.464	<0.001
DKA at presentation	3.991	0.118	0.343
Time at <3.0 mmol/l (<54 mg/dl)	2.251	0.07	0.636
Time in range, 3.9–10.0 mmol/l (70–180 mg/dl)	0.318	−0.038	0.906
Time at >10.0 mmol/l (>180 mg/dl)	0.403	0.046	0.884
CV	0.416	−0.225	0.152
Path length			0.017^a^
Sex	0.223	0.107	0.345
Age	0.037	0.032	0.786
Type 1 diabetes duration	0.009	−0.158	0.280
DKA at presentation	0.226	0.193	0.094
Time at <3.0 mmol/l (<54 mg/dl)	0.083	0.397	0.002
Time in range, 3.9–10.0 mmol/l (70–180 mg/dl)	0.016	−0.065	0.813
Time at >10.0 mmol/l (>180 mg/dl)	0.022	−0.006	0.981
CV	0.030	0.052	0.769

^a^For overall model

*p*≤0.05 was considered significant

#### Probe stage: spatial memory retrieval

During the probe stage, participants with type 1 diabetes spent a median of 59.3 (IQR 37.2–74.5) per cent of the time in the correct quadrant compared with a median of 45.9 (IQR 24.5–69.9) per cent for their healthy control counterparts (*p*=0.055).

The performance at this stage of the vMWMT was significantly linked to nocturnal glucose levels from the previous night among the participants with type 1 diabetes (ESM Table 1). Specifically, time spent in the correct quadrant was inversely correlated with increased time spent in severe hyperglycaemia (*r*=−0.262, *p*=0.026), and with lower nocturnal minimal glucose levels (*r*=−0.337, *p*=0.007). A sex-stratified analysis revealed no statistically significant differences between boys and girls in the probe trial performance or its associations with nocturnal glucose patterns. No statistical significance was found between participants with type 1 diabetes and control participants: median 62.9 (IQR 44.7–74.7)% vs median 46.7 (IQR 30.0–69.5)%, respectively (*p*=0.150) for the boys; and median 48.0 (IQR 32.3–66.4)% vs median 31.3 (IQR 23.0–66.5)% (*p*=0.310) for the girls.

## Discussion

Glycaemic excursions affect cognitive function among adolescents with type 1 diabetes. This study is the first to use a virtual spatial navigation task to explore the relationship between diabetes-related factors and spatial cognitive performance in adolescents with type 1 diabetes. The well-documented sex difference [18] in platform latency, with healthy boys exhibiting shorter times than healthy girls, was also observed in adolescents with type 1 diabetes in the present study. Motor control that was assessed in the visible stages was inferior in adolescents with type 1 diabetes compared with healthy control participants. Notably, spatial learning and memory assessed in the hidden stages part of the evaluation were comparable between the groups. However, longer diabetes duration and greater glycaemic variability predicted worse performance: specifically, each additional year of type 1 diabetes corresponded to a 0.464 s increase in task completion time. Increased time spent in marked hypoglycaemia during the 2 weeks before conducting the task and, particularly during the night prior to testing, were linked to poorer performance and longer path lengths. Our findings underscore the detrimental impact of glycaemic fluctuations on cognitive function during childhood and adolescence.

The visible stages of the vMWMT assess motor-related functions, such as visuomotor integration, movement initiation and motor planning. Boys with type 1 diabetes showed delayed movement initiation and took longer to reach the platform compared with healthy control participants, suggesting inefficiencies in both initiating and sustaining goal-directed motor execution. Moreover, girls with type 1 diabetes were slower to initiate movement and to complete the task compared with healthy girls, indicating broader impairments in sensorimotor processing. Since path length did not differ between the groups, those delays likely reflect slower motor responses rather than impaired navigation. Boys consistently outperformed girls across both the study and control groups, highlighting sex-specific differences in motor function. Given that diabetes-related characteristics were generally similar between sexes, with the exception of higher glycaemic variability in girls, these results suggest that type 1 diabetes may subtly affect motor control, with girls potentially more vulnerable due to sex-specific factors or increased glycaemic variability.

Indeed, accumulating evidence in the literature supports the existence of sex-specific differences in type 1 diabetes characteristics. Diabetes-related disruptions in sensorimotor brain regions have been shown to impair motor performance, aligning with the observed inefficiencies in boys and broader impairments in girls [19]. Deficits in psychomotor speed and visuomotor integration in children with type 1 diabetes, especially in girls, have been reported [20], as was slowed growth in grey and white matter regions involved in motor function [21]. Earlier studies have also identified greater visuomotor integration deficits in girls with type 1 diabetes [22], with altered motor cortex dynamics in boys that may reflect compensatory mechanisms [23], as well as sex-specific differences in neural activation during cognitive tasks in patients with type 1 diabetes [24]. Taken together, these findings underscore the importance of considering sex-based neurodevelopmental trajectories when evaluating motor outcomes in children with type 1 diabetes.

The hidden platform spatial learning stages assess an individual’s ability to learn and remember the location of an unseen goal by integrating spatial memory, attention and higher-order cognitive mapping [25]. At this stage, the performance of boys with type 1 diabetes was comparable to that of healthy peers in initiating movement and locating the hidden platform. Interestingly, they exhibited a shorter path length, which may reflect individual characteristics unrelated to type 1 diabetes. The possibility that children with type 1 diabetes may develop compensatory cognitive mechanisms that paradoxically enhance spatial learning is an intriguing hypothesis. This speculation is further supported by neuroimaging evidence indicating increased brain activation consistent with plasticity-driven adaptations to chronic stress [25]. Spatial learning abilities in girls with type 1 diabetes appeared largely preserved, as performance was comparable with that of healthy control girls in the current study.

The interplay between glycaemic control and spatial memory in type 1 diabetes is complex and not yet fully elucidated. While preclinical studies suggest that chronic hyperglycaemia impairs spatial memory and that insulin may mitigate this effect in murine models [9, 26], human data show mixed results. One large cohort study found that individuals with type 1 diabetes and lower mean HbA_1c_ levels performed better in cognitive tasks than those with higher HbA_1c_ levels [27]. However, our findings did not establish any direct link between HbA_1c_ levels and spatial task performance, indicating that, in the current era of CGM, patients may exhibit improved HbA_1c_ control but that glycaemic variability still poses a risk. In line with this speculation, we found that recent glycaemic variability and excursions, particularly during the night preceding the task, were linked with poorer outcomes, pointing towards short-term glycaemic instability as affecting brain function [27–29]. Alternatively, our finding that diabetes duration independently predicts poorer spatial navigation performance suggests that the brain is impacted not only by acute glycaemic fluctuations but also by their cumulative effects over time.

Long-term effects of diabetes on the brain have been elucidated through findings from neuroimaging studies. Notably, reductions in total brain, grey and white matter volumes were observed, particularly in regions responsible for sensorimotor integration and higher-order cognition, with these changes strongly associated with chronic hyperglycaemia and glycaemic variability [30, 31]. Even in early stages of the disease, lower grey matter volumes in specific cortical areas have been linked to elevated HbA_1c_ levels and lower IQ scores [32]. In addition to cortical changes, structural alterations have been reported in subcortical regions, such as the putamina and thalami, independent of vascular abnormalities [22], while combined structural and functional abnormalities in the midbrain, thalamus and cerebellum further support the notion of widespread diabetes-related neural disruption in children [21]. Among the clinical characteristics we examined, weight status as reflected by the BMI *z* score emerged as a contributor to poorer performance in the vMWMT, with higher BMI *z* scores associated with prolonged time to platform.

Obesity and associated insulin resistance have been reported to contribute to cerebrovascular alterations and cortical thinning, which are also linked to poorer cognitive performance [33]. Notably, increased adiposity has been reported as being more prevalent in the female sex in those with type 1 diabetes [34], underscoring the complex sex-specific manifestations of the disease. A comprehensive investigation incorporating specific cognitive function assessments, CGM, body composition assessment and advanced neuroimaging may offer greater insight into the underlying neural mechanisms affected by diabetes.

In the final stage of the vMWMT, which assesses spatial memory, the negative impact of both hypoglycaemic and hyperglycaemic excursions during the preceding night indicates that recent glycaemic fluctuations may compromise memory retrieval. Severe hyperglycaemia may induce glucotoxicity in selective neurons involved in cognitive processing, with hippocampal neurons having been identified as particularly vulnerable to hyperglycaemia, and with proposed mechanisms including mitochondrial dysfunction and inflammatory responses [35]. Disruption of sleep architecture may also contribute to diminished cognitive performance on the subsequent day. MacAulay et al observed that adolescents with elevated HbA_1c_ levels indicative of chronic hyperglycaemia had increased stage N3 sleep and decreased rapid eye movement (REM) sleep [36]. Adolescents with type 1 diabetes experiencing nocturnal hyperglycaemia had higher sleep onset latency, increased light sleep percentage and higher arousal index compared with control individuals, indicating that hyperglycaemia leads to fragmented and less restorative sleep [37]. A meta-analysis further linked recurrent severe hypoglycaemia, particularly during sleep, to memory and learning deficits in this population [38]. Our findings, along with such evidence linking nocturnal glycaemic levels to sleep quality, strongly support the need to maintain stable overnight glucose management in youth with type 1 diabetes and emphasise its critical importance for overall health.

### Limitations of the study

This study has several limitations. First, as a single-centre investigation involving adolescents with relatively well-controlled type 1 diabetes, the findings may not be generalisable to broader populations with greater socioeconomic, ethnic or clinical diversity. The relatively good glycaemic control of our participants may limit generalisability to the broad population with type 1 diabetes and attenuate potential associations with hyperglycaemic exposure and DKA. Since the SEP data were incomplete for the control group and given that the overall reported prevalence of attention deficit hyperactivity disorder (ADHD) was low in both groups, we did not adjust the study outcomes for these variables, and future studies should further examine their potential impact. Second, the cross-sectional design precludes conclusions about causality between glycaemic control and cognitive outcomes. Finally, important factors known to affect cognitive function, such as sleep quality, psychological stress and physical activity, were not directly measured and should be addressed in future research.

### Conclusions

This study demonstrates the utility of vMWMT analysis in elucidating the impact of type 1 diabetes on cognitive function in adolescents and reveals sex-based differences and associations with glycaemic control. While spatial memory retrieval remained intact, longer diabetes duration and frequent severe episodes of hypoglycaemia in the 2 weeks preceding vMWMT analysis were linked to poorer visuospatial task performance. Nocturnal glycaemic excursions were also adversely related to task performance. Our findings emphasise the need for sex-specific considerations in diabetes management and underscore the importance of maintaining stable glucose levels and preventing severe hypoglycaemia to protect cognitive function. Further research is warranted to explore the cognitive outcomes of adolescents with type 1 diabetes using advanced glucose management technologies.

## Supplementary Information

Below is the link to the electronic supplementary material.

ESM Table (PDF 188 KB)

## Data Availability

The data that support the findings of this study are not openly available due to reasons of sensitivity and are available from the corresponding author upon reasonable request.

## Abbreviations

AID: Automated insulin delivery
CGM: Continuous glucose monitoring
DKA: Diabetic ketoacidosis
GMI: Glucose management indicator
MDI: Multiple daily injections
SEP: Socioeconomic position
vMWMT: Virtual Morris water maze task
