# Supplementary material for: The virtual Morris water maze for cognitive function assessment in adolescents with type 1 diabetes
Source: Diabetologia. 2025 Nov 17;69(2):491–503. doi: 10.1007/s00125-025-06598-x (PMC12779742; doi:10.1007/s00125-025-06598-x)
Supplement: Supplementary file 1 — ESM Table (PDF 188 KB) [file 125_2025_6598_MOESM1_ESM.pdf]

ESM Table 1 Correlation between task performance parameters and other variables in the hidden stages and probe stage

Task Parameter	Variable Category	Variable	r	*p* value
**Hidden stage**				
**Time to first move**	Demographic and Clinical Characteristics	BMI z scores	0.210	0.072[p]
		T1D duration	0.096	0.467
		Age at diagnosis	0.025	0.833[p]
	Glycaemic Measures (HbA_{1c}, GMI)	HbA_{1c} (mean)	0.019	0.871
		HbA_{1c} (SD)	0.092	0.433
		HbA_{1c} at current visit	0.143	0.228
		HbA_{1c} at onset	−0.108	0.378[p]
		GMI	−0.007	0.956[p]
	CGM Metrics	Coefficient of variation	−0.086	0.484[p]
		Time CGM active	−0.170	0.163[p]
		Time in range, 70–180 mg/dL [3.9–10.0 mmol/l]	0.014	0.905
		Time >250 mg/dL [>13.9 mmol/l]	0.041	0.731
		Time <54 mg/dL [<3.0 mmol/l]	0.035	0.769
	Nocturnal glucose metrics in the previous night	Minimum glucose	0.267	**0.035***
		Maximum glucose	0.092	0.472

		Time in range, 70–180 mg/dL [3.9–10.0 mmol/l]	−0.048	0.711
		Time <54 mg/dL [<3.0 mmol/l]	−0.284	0.240
		Time >180 mg/dL [>10.0 mmol/l]	0.140	0.273
**Time to platform**	**Demographic and Clinical Characteristics**	BMI z scores	0.281	**0.015** [p]
		T1D duration	0.395	**0.002**
		Age at diagnosis	0.012	0.921 [p]
	**Glycaemic Measures (HbA_{1c}, GMI)**	HbA_{1c} (mean)	0.109	0.353
		HbA_{1c} (SD)	−0.007	0.950
		HbA_{1c} at current visit	0.194	0.099
		HbA_{1c} at onset	−0.153	0.210 [p]
		GMI	0.099	0.432 [p]
	**CGM Metrics**	Coefficient of variation	0.075	0.544 [p]
		Time CGM active	−0.255	**0.034** [p] *****
		Time in range, 70–180 mg/dL [3.9–10.0 mmol/l]	−0.100	0.402
		Time >250 mg/dL [>13.9 mmol/l]	0.129	0.282
		Time <54 mg/dL [<3.0 mmol/l]	0.175	0.141
	**Nocturnal glucose metrics**	Minimum glucose	0.214	0.092

	**the previous night**			
		Maximum glucose	0.146	0.254
		Time in range, 70–180 mg/dL [3.9–10.0 mmol/l]	0.042	0.747
		Time <54 mg/dL [<3.0 mmol/l]	−0.178	0.162
		Time >180 mg/dL [>10.0 mmol/l]	0.172	0.179
**Path length**	**Demographic and Clinical Characteristics**	BMI z scores	0.107	0.362 [p]
		T1D duration	−0.047	0.726
		Age at diagnosis	−0.048	0.686 [p]
	**Glycaemic Measures (HbA_{1c}, GMI)**	HbA_{1c} (mean)	0.109	0.354
		HbA_{1c} (SD)	−0.295	**0.011**
		HbA_{1c} at current visit	0.092	0.440
		HbA_{1c} at onset [p]	−0.055	0.652 [p]
		GMI [p]	0.104	0.412 [p]
	**CGM Metrics**	Coefficient of variation [p]	0.253	**0.037 [p] ***
		Time CGM active	−0.182	0.135 [p]
		Time in range, 70–180 mg/dL [3.9–10.0 mmol/l]	−0.143	0.230
		Time >250 mg/dL [>13.9 mmol/l]	0.120	0.316
		Time <54 mg/dL [<3.0 mmol/l]	0.394	**<0.001**

	**Nocturnal glucose metrics in the previous night**	Minimum glucose	0.085	0.508
		Maximum glucose	0.133	0.300
		Time in range, 70–180 mg/dL [3.9–10.0 mmol/l]	0.063	0.622
		Time <54 mg/dL [<3.0 mmol/l]	−0.012	0.925
		Time >180 mg/dL [>10.0 mmol/l]	0.119	0.352
**Probe Stage**				
**Time in the correct quadrant**	Demographic and Clinical Characteristics	BMI z-scores	−0.142	0.226[p]
		T1D duration	−0.155	0.240
		Age at diagnosis	−0.050	0.673
	Glycaemic Measures (HbA_{1c}, GMI)	HbA_{1c} at current visit	−0.208	0.078
		HbA_{1c} at onset	0.122	0.317[p]
		GMI	−0.162	0.196[p]
	CGM Metrics	Coefficient of variation	−0.089	0.471[p]
		Time CGM active	−0.227	0.060[p]
		Time in range, 70–180 mg/dL [3.9–10.0 mmol/l]	0.191	0.109
		Time >250 mg/dL [>13.9 mmol/l]	−0.262	**0.026***
		Time <54 mg/dL [<3.0 mmol/l]	−0.131	0.272

	Nocturnal glucose metrics in the previous night	Minimum glucose	−0.337	**0.007**
		Maximum glucose	−0.166	0.193
		Time in range, 70–180 mg/dL [3.9–10.0 mmol/l]	−0.027	0.836
		Time <54 mg/dL [<3.0 mmol/l]	0.258	**0.041***
		Time >180 mg/dL [>10.0 mmol/l]	−0.098	0.445

Spearman/Pearson correlation analyses between probe stage, hidden stage and other variables. Pearson's correlation was used for variables with normal distribution (marked with ^{p}), whereas Spearman's rank correlation was used for variables with skewed distribution. BMI, body mass index; GMI, glucose measurement indicator; CGM, continuous glucose monitoring; HbA_{1c}, haemoglobin A1c. A *P* value of ≤0.05 was considered significant. **Bold** indicates significance.
* Not statistically significant after Bonferroni correction
